# An Account of the North Kensington Friendly Workers among the Poor

**Published:** 1892-09-03

**Authors:** C. Furley-Smith


### THE CURE OF POVERTY,

### AN ACCOUNT OF THE NORTH KENSINGTON FRIENDLY WORKERS AMONG THE POOR

—(*continued from page* 368).

By Mrs. C. Furley Smith.

It is desirable then that all who desire to ameliorate the condition of the poor should work together. By this means it would be easier to give to every form of poverty the help it most required, and to obtain this from the individuals or the community on whom it had most claim. To this end the following representative committee was elected.

*Chairman*—Mr. Henry C. Burdett.
*Treasurer*—Sir Roper Lethbridge, K.C.I.E., M.P.
*Secretary and Agent*—Mr. Donald Mackenzie.
Rev. J. A. Betts (St. Columb's), Church of England.
Brigade-Surgeon A. D. Campbell (Presbyterian Church).
Mr. J. Foster (Lancaster Road Congregational Church).
Captain W. T. Foster, 36, Burlington Road, W.
Mr. F. C. Frye, L.C.C., 19, Colville Mansions.
Rev. R. Harris (Jewish Minister).
Captain James, R.E., L.C.C., 19, Lexham Gardens.
Mr. J. H. Pollen (St. Vincent de Paul), Roman Catholic.
Rev. J. Stevens (Lancaster Road Congregational Church).
Rev. Father Sylvester (St. Mary-of-the-Angels), Roman Catholic.
Rev. Canon Trench (All Saints', Kensington Park), Church of England.
Rev. J. Tuckwell (Westbourne Grove Chapel).
Very Rev. Father Wyndham (St. Mary-of-the-Angels), Roman Catholic.

It will be said: "All those gentlemen could avail themselves of charitable agencies, congregational or other, already in existence; why, then, should they unite to form one more?" Simply because they had command of these agencies. The Association of Friendly Workers did not propose to form another charity; its main object was not the distribution of relief, but to develop to the utmost possible extent the usefulness of the agencies already existing. Was it, then, simply a local Charity Organisation Society? Hardly that. The Charity Organisation Society has done, is doing, good work, but it hardly fills the position sought for by the North Kensington Friendly Workers. This has three main objects:—

1. To know the poor personally and as friends. To win their confidence, so that they will be ready to ask advice as well as help. To be on such terms with them that they shall know where to go in their day of trouble, sure of fair and sympathetic treatment, though not of indiscriminate alms. There are plenty of cases for philanthropic intervention where people do not want alms. Take, for example, people living in insanitary houses. They complain to the landlord or his agent, but nothing is done. As a rule they do not know where to apply next. They do not know the name of the medical officer of health for the district; perhaps they do not even know that there is one; and they do not know how to approach him. If, however, they can apply to the Friendly Workers and explain the matter to the Secretary of the Association, he will lay it before the proper authority, and prompt attention has always been the result. But people would not think of thus laying the case before Mr. Mackenzie if they had not come to know him as a true and reliable friend. To provide such a friend has been the aim of the association.

For the strictly charitable work—that giving to the needy to which the word is generally confined—the names of the committee are sufficient guarantee. Clergymen and laymen of almost every denomination, each with the command of the usual congregational charities, united in the good work. They worked together in true brotherhood. On this point, so often held up as an excuse for not trying to work charitable organisations in union, there was never any difficulty from first to last. It was convenient and fitting that they should be asked to help chiefly those of their own communion, and they were glad to know, through an impartial organisation, of the existence of those who had special claims upon their kindness; while they had the satisfaction of feeling that, as the association visited every poor family in the district, and had the names of all in their books, no one who appealed through it could, by the hypocritical acts so often practised, obtain help from two churches simultaneously. This prevention of the duplication of charities has been one of the most valuable features of the scheme.

I do not wish to dwell in any detail upon the individual members of the committee. Of the chairman, Mr. Burdett, I will mention only this among his many useful qualities—that when he wants a thing done, he has the faculty—a faculty amounting almost to genius, for finding the man to do it. In this case the man to do it was Mr. Donald Mackenzie, the Secretary of the Association. In his speech at the Conference, held in All Saints' Schools, Mr. Burdett said that his scheme required the supreme devotion of a few men and women. In at least one man he found that supreme devotion. At first without any remuneration whatever, afterwards at a salary which it would be absurd to talk of as payment for his labours, Mr. Mackenzie has lived for the Association of Friendly Workers. His personal efforts, his personal influence, his personal acquaintance with the people he was dealing with, have been important factors in the success of the Association. Without disparaging in the least the work of his fellow-labourers, we may admit the pre-eminent value of Mr. Mackenzie's, for he pre-eminently has given himself to spend and be spent in the cause of the North Kensington poor.

It is true that in many cases people do thus give themselves with results quite disproportionate to the sacrifice involved. Perhaps that is because they feel it to be a sacrifice. Love for the work which he had undertaken is one of the secrets of Mr. Mackenzie's success. Other elements are these: He is a man. The charitable worker is commonly a lady, often a young lady with but the vaguest and most external knowledge of the problems she has to deal with in every poverty-stricken home she visits. Do not let me be supposed to scorn the lady visitor. Especially if she be a middle-aged woman into whose own experience love and grief and anxiety have come, she can give endless help and comfort to overburdened mothers, wise counsel and sympathy to working girls, who are resentful of supposed injustice, impatient of the gentlest restraint. Even the young girl who withdraws herself now and then from her own amusements and vague, rose-hued dreams to visit gloomy homes in narrow streets, does good in at least showing that she holds herself a sister of the poor; although these, with the instinctive delicacy and the respect for the rights of youth and joy which mark them, do not tell her half their troubles. But to deal with men, a man is required. He can be supposed to know the trials and temptations of other men; and on the strength of this understanding he can speak straightforwardly and fearlessly of faults and failings by which poverty has been invited to take up its abode, and by the force of his stronger personality he can effect a reformation which all the mild feminine persuasions in the world would fail to do.

But the man who does this must be—a gentleman. It is hard to be compelled to use that word—

> Profaned by every charlatan,
> And soiled by all ignoble use, but I know no other to express what I mean. The race of Bumble is not yet extinct in the land, and the poor loathe Bumble. Political enthusiasts declare that it is because he will lose his vote if he accepts parochial relief that the working man will come nigh starvation before he asks for it. As a rule, it is only when he is tolerably prosperous that the working man clings to his vote. After all, you can't eat a vote, and in these days it is difficult even to drink it. And even if the political enthusiasts are right about the working man, how will their theory explain the equal, the often more intense repugnance of women, who have no votes, to asking aid from the union? The vote has very little to do with it; it is loathing of the inquisition of Bumble. Bumble is often a respectable citizen enough in his way, sometimes well meaning, not wholly destitute of human kindness behind his imperial manner; but he is not a gentleman. He knows the world, or thinks he does; he knows, too, the poor and their ways, or thinks he does—

> "Take my word for it, Sammy, the poor in a lump is bad"—

and by way of proving that knowledge he often conducts his inquiries into the cases that come before him with an insolence, a brutality of method which makes poverty indeed seem a crime and helplessness a disgrace. Any society which trusts its active work to Bumble throws away half its power for good. The North Kensington Association of Friendly Workers avoided Bumble; it put the conduct of inquiries and the bestowal of help into the hands of an intelligent and educated gentleman—a man who for his devotion to the welfare of the poor, for his honest consideration for their rights and feelings, for his conscientious inquiry into their circumstances, and the best methods of helping them, was worthy of the trust reposed in him alike by those who desired to help and those who sought to be helped. And last, but not least, a man who could become morally strong enough to rise beyond the reach of being disheartened and hindered in his course, by the well-intended, but at the same time, thoroughly selfish advice of friends, who would appear to approve only of that kind of charity (if such there can be), which costs nothing and calls for little or no self-denial to practice.

So much for the man; now let us speak of the methods.

*(To be continued.)*

